# VIA Family—a family-based early intervention versus treatment as usual for familial high-risk children: a study protocol for a randomized clinical trial

**DOI:** 10.1186/s13063-019-3191-0

**Published:** 2019-02-08

**Authors:** Anne D. Müller, Ida C. T. Gjøde, Mette S. Eigil, Helle Busck, Merete Bonne, Merete Nordentoft, Anne A. E. Thorup

**Affiliations:** 1 Research Unit, Child and Adolescent Mental Health Center, Lersø Parkallé 107, 1 th, 2100 Copenhagen, Capital Region of Denmark Denmark; 2 Mental Health Center, Copenhagen, Capital Region of Denmark Denmark; 3 Municipality of Frederiksberg, Family Department, Copenhagen, Denmark; 4 University of Copenhagen, Institute for Clinical Medicine, Faculty of Health Science, Copenhagen, Capital Region of Denmark Denmark

**Keywords:** Familial high-risk, Offspring, Schizophrenia, Bipolar disorder, Recurrent depression, Multidisciplinary, Early intervention, Child mental health, Parental training, Family-based intervention

## Abstract

**Background:**

Children born to parents with a severe mental illness, like schizophrenia, bipolar disorder, or major recurrent depression, have an increased risk of developing a mental illness themselves during life. These children are also more likely to have developmental delays, cognitive disabilities, or social problems, and they may have a higher risk than the background population of experiencing adverse life events. This is due to both genetic and environmental factors, but despite the well-documented increased risk for children with a familial high risk, no family-based early intervention has been developed for them. This study aims to investigate the effect of an early intervention that focuses on reducing risk and increasing resilience for children in families where at least one parent has a severe mental illness.

**Methods/design:**

The study is a randomized clinical trial with 100 children aged 6–12 with familial high risk. It is performed in the context of the Danish health-care system. Families will be recruited from registers or be referred from the primary sector or hospitals. The children and their parents will be assessed at baseline and thereafter randomized and allocated to either treatment as usual or VIA Family. The intervention group will be assigned to a multidisciplinary team of specialists from adult mental health services, child and adolescent mental health services, and social services. This team will provide the basic treatment elements: case management, psychoeducation for the whole family, parental training, a safety plan, and potentially an early intervention if the child has mental problems. The study period is 18 months for both groups, and all participants will be assessed at baseline and after 18 months. The primary outcome measure will be daily functioning of the child, and the secondary measures are the psychopathology of the child, days of absence from school, family functioning, child’s home environment, and parental stress.

**Discussion:**

This study is to our knowledge the first to explore the effects of a multidisciplinary team intervention that provides an intensive and flexible support to match the families’ needs for children with a familial high risk for severe mental illness. The study will provide important knowledge about the potential for increasing resilience and reducing risk for children by supporting the whole family. However, a longer follow-up period may be needed.

**Trial registration:**

ClinicalTrials.gov, NCT03497663. Registered on 13 April 2018.

**Electronic supplementary material:**

The online version of this article (10.1186/s13063-019-3191-0) contains supplementary material, which is available to authorized users.

## Background

Children born to parents with a severe mental illness (SMI), such as schizophrenia spectrum disorders, bipolar affective disorder, or major recurrent depression, are a group of familial high-risk children overlooked by the mental health service system and the social services of their municipality [1–3]. Research shows that these children are vulnerable biologically, genetically, as well as socially, and that they have an increased risk of developing a mental illness themselves [4]. More than half of them will at some point in life develop a mental illness and about one third will develop an SMI*.*

In the mental health service system, there is a growing awareness of the need to develop preventive strategies as an early intervention for mental illness. However, few concrete ideas have been identified and investigated. An early intervention for children with a familial high-risk of SMI could consist of providing better resources to the family, and psychoeducation and parental training—all initiatives that are supposed to strengthen the normal and healthy development of the child and prevent later mental illness. This strategy is widely recommended in the international literature [5, 6].

### High-risk studies and early risk factors

Over recent decades, several familial high-risk studies, i.e., cohort studies of individuals born to parents with SMI, have been conducted. These studies have documented that the increased risk of developing mental illness is caused by complex interactions between genetic and environmental factors [7–9]. This has especially been shown for schizophrenia [10–13], but also for affective disorders, i.e., bipolar disorder [14, 15], and major recurrent depression [16]. There is growing evidence that on a group level, these three diagnostically different disorders have a considerable phenomenological, biological, and genetic overlap [4, 17] . Research has also shown that children born to parents with an SMI more often display a series of the early signs of mental vulnerability or delayed or disturbed development during childhood [18–20]. These signs are understood as early risk factors for the development of a mental illness later in life. Prenatal risk factors include infections during pregnancy, unwanted pregnancy, stress and trauma during pregnancy, and obstetric complications [5, 21]. Examples of risk factors during childhood are different kinds of childhood traumas such as child neglect, physical abuse, emotional abuse, sexual abuse, and bullying [22]—all of which may have a serious, negative impact on child development [5, 23]. Moreover, there are a number of social early risk factors, such as having a mother diagnosed with schizophrenia, which may increase the risk of living in a dysfunctional family [23], with a single parent [24] or a parent with poor parenting skills [25], or in poor socioeconomic conditions [26]. It is important to mention that some children of parents with an SMI will not be influenced by these risk factors, while others will be affected by several.

### Neurodevelopmental perspective

Studies have shown that familial high-risk children born to parents with an SMI are vulnerable in several aspects [27–31]. Familial high-risk children are more likely to have unspecific mental health problems like anxiety, autism spectrum symptoms, or subtle psychosis-like symptoms called psychotic-like experiences, such as brief or transient hallucinations or delusion-like convictions. They may also show delayed motor or language development or cognitive disabilities, like attention deficits or problems with working memory or impulse control [10, 27, 32–34]. Furthermore, behavioral patterns, like poor emotional regulation (i.e., the ability to regulate and adapt emotions and impulses to the given situation) [35], affective control [36], or social adaptation, may also be affected in familial high-risk children [5, 19].

This increased mental vulnerability of the children is best understood from a neurodevelopmental perspective, which is to say that the child’s normal developmental process is influenced by interacting aspects of genetic disposition and environmental factors broadly speaking, including, for example, insufficient parental support and stimulation, increased risk of childhood trauma, unstable living conditions, and poor social status [7, 23].

Mental illness can, thus, be understood as neurodevelopmental deviations that start very early in life and are influenced by risk factors as well as resilience factors throughout the individual’s life [21, 37, 38].

### Parental perspective

Parents with an SMI often lack resources in several aspects of life—material, financial, psychological, or social. It can, for some, be a very demanding task to provide their children with the necessary level and amount of support, care, and stimulation [39]. Further, they may not always have the energy or overview to navigate the complicated public health system and social services, so that the provision of help or support may be uncoordinated or random. The level of conflicts and disagreement in the families may be high and access to resilience-providing factors, like sound social relations or leisure activities, may be limited. Some children are overinvolved in their parent’s symptoms, such as delusions, hallucinations, or negative and depressive symptoms, which sometimes leads to over-responsible children, also called young carers, i.e., children doing tasks that should be done by an adult [40]. All these factors may increase the child’s risk of developing a mental illness later in life [41, 42].

However, it is important also to mention that parents who, at some time in their life, have suffered from an SMI can do very well in terms of parenting, especially if relevant and specialized support is available [43].

### Early preventive interventions

This evidence has led to increased interest in investigating the potential of intervening at an early stage, i.e., before the child is diagnosed with a mental illness for the first time or before the problems get too big and complex. This is widely recommended in the international research literature but has been tested systematically only in a very few instances and not before in Denmark [5, 6]. The hypothesis is that by providing a very early intervention it will be possible to protect the most vulnerable individuals against the known risk factors (e.g., childhood trauma or some of the consequences of cognitive disabilities) and strengthen the individual’s resilience by enhancing protective factors (e.g., social relations or recreational activities). This very early intervention will inhibit or diminish the cascade effect, which is seen when several risk factors over time influence an individual’s development. This may be true not only for individuals who are born with familial high-risk but for people in general [44–47].

### International studies on early preventive interventions

There are a few papers investigating the overall effect of an early, preventive approach, but in general effect sizes are small and there is still a lack of knowledge of many aspects [47, 48]. The Canadian study Families Overcoming Risks and Building Opportunities for Well-Being (FORBOW) led by Prof. Uher aims at providing an intervention in the form of cognitive therapy and psychoeducation for families with an SMI of any kind, but no results are available yet [29]. The idea is that the intervention is “low-burden, low-risk,” i.e., not risky for the participants, as, for example, medication could be, and it is directed against a concrete and currently existing problem or situation that it seems relevant to alleviate or solve for the family here and now. Other initiatives [5] emphasize the importance of parental skills training and support, psychoeducation, reducing the effects of cognitive disabilities, and providing practical, financial, and legal assistance.

### Development of intervention and pilot testing

Based on the above mentioned evidence from the existing literature combined with clinical experience from the Danish High-Risk and Resilience Study VIA 7 [31, 49] (conducted by the last author) and results from qualitative studies including focus group interviews by our own research group, we have proposed a model for a specialized intervention for this specific group of children. Based on this model, a manual for the intervention was developed and pilot tested by the intervention team in the pilot phase of the study. The pilot study consists of two volunteer families, who were included 5 months prior to the start of the study period and who agreed to give feedback to the team during the 18 months of the intervention.

## Methods/design

This paper was written in line with the explanation and elaborations relating to the guidance for protocols of clinical trials in the 2013 SPIRIT guidelines (Standard Protocol Items: Recommendations for Interventional Trials). The SPIRIT figure (schedule of enrolment, interventions, and assessments; Fig. 2), SPIRIT checklist (Additional file 1), and the World Health Organization Trial Registration Data Set (Additional file 2) were used.

### Trial design

The VIA Family study is a two-armed parallel-group randomized clinical trial testing for superiority of the VIA Family group. The treatment allocation ratio is 1:1, stratified exclusively by diagnostic group. The allocation will be executed in a preset concealed block size order.

### Objectives

The main objective of the trial is to test the VIA Family intervention against treatment as usual (TAU). Currently, there is no specific treatment or support for familial high-risk children born to parents with an SMI in Denmark.

#### Research question and hypothesis

The research question for this project is

- to investigate the effect of VIA Family, a multidisciplinary and family-based intervention for children born to a parent with an SMI, with a series of preset outcome measures

The hypothesis is that by providing such children and their families with an integrated, multidisciplinary, specialized, non-stigmatizing, and family-based intervention, it will be possible

- to improve the child’s daily functioning, including a reduction in the number of days absent from school
- to reduce the magnitude of the child’s mental problems and symptoms of psychopathology
- to improve the general functioning of the family
- to relieve the total burden experienced by family members and increase well-being

Over a longer perspective (5–10 years), we hypothesize that VIA Family will:

- reduce the prevalence and severity of mental illness among the children
- enable parents to develop sufficient parenting skills to reduce the possibility that their children will be referred to institutional upbringing or foster care

### Participants, interventions, and outcomes

#### Study setting

The VIA Family study is a close collaboration between the Child and Adolescent Mental Health Center, Capital Region of Denmark, the Adult Mental Health Center Copenhagen, Capital Region of Denmark, and the local social services. Families are recruited from the municipalities of Frederiksberg and Copenhagen. The intervention sessions are held in a single center in Copenhagen in a building away from all hospitals and in a different location from the research team doing the assessment at baseline and follow-up.

#### Eligibility criteria

The inclusion criteria are:

1. Families in the Frederiksberg or Copenhagen municipalities who have at least one child aged 6–12. Families living in Frederiksberg are prioritized. Further, at least one of the parents must have a diagnosis of an SMI, which is defined as schizophrenia spectrum disorder (ICD-10 codes F20, F21, F22, or F25, or ICD-8 codes 295, 297, 298.29, 298.39, 298.89, 298.99, or 301.83), bipolar affective disorder (ICD-10 codes F30 or F31 or ICD-8 codes 296.19 or 296.39), or recurrent moderate or severe depression (ICD-10 codes F 33.1, F33.2, or F 33.3 or ICD-8 codes: 296.0, 298.0 or 300.4). The diagnoses will be confirmed by the diagnostic interview Schedules for Clinical Assessment in Neuropsychiatry (SCAN) [50] at baseline.
2. For those recruited from the register sample, the parent with an SMI diagnosis must have had at least one in- or outpatient contact with the mental health system within the lifetime of the child.

The exclusion criteria are:

1. Parents who do not speak and understand enough Danish to be able to give informed consent for their own participation and for the child’s participation.
2. If all family members are currently engaged in an intensive family intervention program addressing parental functioning and child development.

#### Criteria for discontinuing the intervention

If a family withdraws informed consent, they will be excluded from the study. All participants will still be asked to participate in the follow-up interviews.

#### VIA Family intervention

**Fig. 1:**
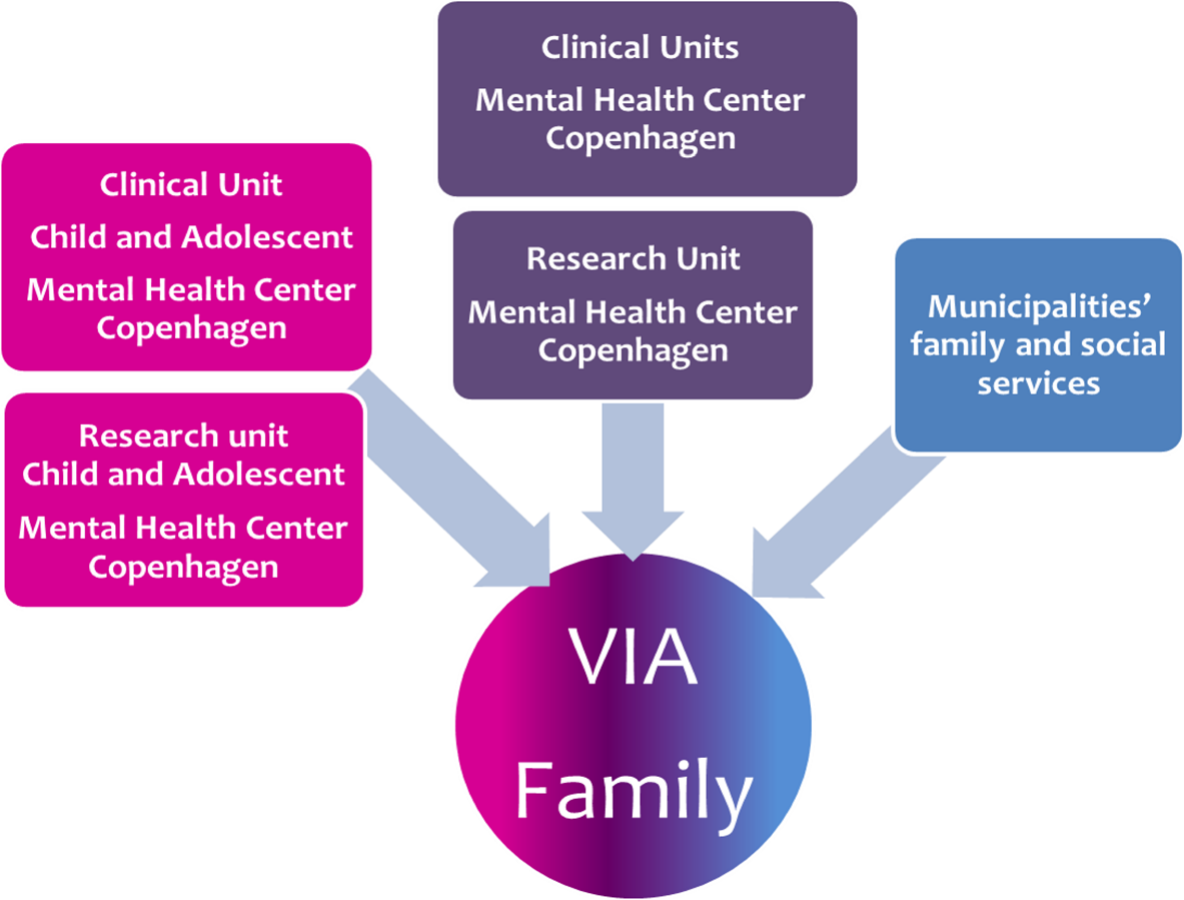
Model for multidisciplinary resources in the team

The families, who by randomization are assigned to a VIA Family team, are offered contact with a specialized multidisciplinary team. This team’s competences include knowledge and experience of the mental health system and municipality social services, allowing them to achieve a high degree of cross sectional synergy to meet the families’ needs as much as possible (Fig. 1). A team consists of five members:

- a child and adolescent psychiatrist or physician who has experience with providingchild and adolescent mental health services
- a psychologist who has experience with providing child and adolescent mental health services
- a nurse with clinical experiences from adult mental health services
- a social worker who has worked with families in the municipality
- a family counsellor or family therapist who has worked with children in the municipality

The trial is a pragmatic trial, since we know that the target families’ needs and problems are very diverse and will vary over time. During the first two meetings, two team members will collaborate with the family in trying to uncover what kind of problems or unmet needs the child or the family may experience. In cooperation with the family, a problem-solving plan will be produced that focuses on the issues that the family has described. VIA Family is based on a series of basic elements and a number of additional options can be included if they are suitable for the family’s subjective preferences.

The basic elements in VIA Family are:

- *Case manager.* A case manager will be designated for the whole family. They will be easily accessible and can offer home visits if relevant. The case manager can help to coordinate appointments, will participate in meetings, and will give information and advice concerning possible support from the municipality. The case manager is generally available for the family for questions, worries, or problems of any kind and will guide the family to the right resources if the problem is outside the capacity of the VIA Family team. The case manager will stay in touch with the family when the family is not actively working with the team, e.g., by sending text messages or making phone calls.
- *Psychoeducation.* This focuses on what it is like to be part of a family where one of the parents has an SMI. This may also be relevant for those children who do not live with the ill parent. The case manager (often together with another team member) will provide a specialized psychoeducational course that is based on the family’s current situation and relevant aspects of the parent’s illness status. The course consists of approximately 6–8 sessions, which include all family members. There is a planning phase of 1–3 sessions with the parents followed by 1–3 sessions where the children are present. The course ends with an evaluation session with the parents, which includes considering the next issue for the family to work with. The team has written and collected relevant Danish-language material including books and films that can be used.
- *Parental training and support for the parenting role.* The parents are offered parental training based on the evidence-based Positive Parenting Program (Triple P) [51–53], with intensity and content according to their specific needs. Triple P is a multilevel program, meaning that the intensity, content, and administration of the program can be adapted to each family’s specific situation. It starts at a less intensive, general level and can then be unfolded to a more intensive level if needed or if needs change. There are highly specific modules for specific target groups and families and for specific problems.
- *Safety plan and mapping the social network and the social resources of the family*. The safety plan can be used by any family member in an unexpected situation or an acute crisis, e.g., if the family’s plans and routines are not being followed due to the ill parent’s sudden need for psychiatric first aid or hospitalization. The safety plan addresses issues such as who the family members can contact, what to tell the children, and who should take care of them in an acute crisis. It can include resources from the municipality, possibly as a strategy to prevent an acute situation. For the ill parent, meetings about the parent’s mental state can be arranged within a short time (e.g., if there are early signs of exacerbation). Supportive meetings for the other parent are also provided, since being the closest relative can be demanding.

#### Other additional treatment options offered by the VIA Family team

If needed, the team can offer the following treatment elements facilitated by the case manager:

- *Specialized treatment for the child’s (transient or sub-threshold) mental problems or disturbances*. This can include anxiety, obsessive–compulsive disorder, tics, or attention problems. The child and adolescent psychologist and the child and adolescent psychiatrist in the team can provide cognitive therapy or psychoeducation according to Danish national guidelines for treatment (from the Danish Ministry of Health), and also specific cognitive therapy, e.g., for children who have had psychotic-like experiences.
- *Treatment groups for children, parents, and relatives.* The children will be offered a group course of 6–10 meetings with 6–8 other children in a similar situation. Together with a group therapist, they will have the opportunity to talk openly about their thoughts, experiences, and worries, and they can ask questions and listen to the other children’s coping strategies. The parents can simultaneously participate in groups for relatives and parents with a mental illness (parent café), where presentations about relevant topics and networking between the participants will be the main content. Treatment groups are either facilitated by the VIA Family team or by an external partner, to whom the families will be directly referred, if they prefer.
- *Counselling and guidance regarding financial, social, or practical support from the municipality*. This may make it possible for the child to attend some kind of leisure activity, like sports, the scouts, or music. The financial support may pay any extra costs due to child-related problems (e.g., washing and nappies for a child with enuresis, which is very common). The social support may be a supportive adult who can build an independent relationship with the child while also giving the parent a break. The case manager will assist the family in the application process, maybe assisted by the social worker in the team. The municipality will make the final decision regarding any request for support.
- *Information about or supervision by institutions and schools*. This may be about the parent’s mental illness or possible mental problems that the child might have, if the parents agree.
- *Optimization of the ill parent’s treatment and lifestyle.* This will be done in close collaboration with the Adult Mental Health Center. Dialogues focusing on the parenting role in relation to a recovery process could be offered by the case manager.
- *Fast track to mental health services.* The Adult Mental Health Center at Frederiksberg will offer all families in the VIA Family group a fast track for acute psychiatric assessments, which the case manager can refer to whenever relevant. The Child and Adolescent Mental Health Center will also offer a fast track for any acute child psychiatric situation and will provide supervision for more complicated cases concerning the child’s mental health problems or any detected need for further examination and diagnosing.
- *Other help*. Whenever possible, the team will also try to help and assist the family with other matters that are relevant for their well-being and everyday functioning. It can help them to find the right resources needed to solve a problem.

The sequence of engaging in the various elements is: introduction (1–2 sessions), life line and history with both parents (2–4 sessions), psychoeducational course (6–8 sessions), Triple P (3–10 sessions), and groups for both children and parents (8 sessions), followed by a flexible period of up to 16 months of intervention with individual or family meetings, evaluation, termination, and transition to TAU.

The VIA Family team is not based in a hospital. The building has a very non-clinical atmosphere, which is family and child friendly. It can accommodate meetings and confidential conversations and it has a room for group sessions and educational courses. The surroundings are suitable for families with small children too. A small selection of psychoeducational literature, books, and folders are available for free.

#### Monitoring intervention fidelity

To ensure treatment fidelity, intervention team members are all trained and supervised in treatment delivery and the intervention is regularly monitored by the principal investigators of the study group.

The VIA Family intervention is a specialized intervention consisting of different treatment elements, which are offered to all families. Each family will receive an individual package containing different elements and doses of the intervention. To measure treatment fidelity, the intervention team will register every single element of treatment given to a family for later analyses (e.g., how many hours of psychoeducation and which members of the family participated, number of contacts, number of meetings etc.) in the data entry system Research Electronic Data Capture (REDCap) [54]. To ensure some degree of consistency for the VIA Family intervention and to differentiate the experimental treatment from TAU, some criteria for minimum participation in the VIA Family group have been defined: (1) a minimum of 15 contacts with the case manager (at least two contacts with personal attendance), (2) at least one session of psychoeducation, (3) one session to map the safety plan for the family, and (4) one session to address parental training.

#### Treatment as usual

The families who by randomization are assigned to TAU will continue to receive the same kind of help and support as they did before entering the study. TAU is defined as any kind of help and support focusing on high-risk children and parental mental illness. At present, the municipalities and the mental health services do not offer any kind of family-focused intervention addressing parental mental illness that can be compared to the VIA Family program. Moreover, the VIA Family team will work only with the families randomized to VIA Family, thus contamination from TAU to the VIA Family intervention will be very limited. We expect a variation in TAU, as some of the families who are invited to participate will not be involved with the municipality or the mental health system, while other families are.

After the baseline assessment and before randomization, all families (TAU and VIA Family) will receive a brief phone call offering a standardized guide to the psychosocial help and support for high-risk children and their families available within the municipalities (Additional file 3). All families will also be offered verbal feedback on their child’s performance and mental health status after the assessment has been completed.

The families in the VIA Family intervention group will have access to the same help and support available within the municipalities and mental health services as the TAU group, hence TAU is common for both groups. However, we expect a difference in the usage of TAU, because the case manager in the VIA Family intervention group will help the families to access the relevant services in the community. Moreover, some of the services available within TAU and the intervention (e.g., child support groups) have limited capacity and access for participation within TAU but are offered to all families in the VIA Family group. Data on each child’s service use and on municipality involvement and support for the whole family will be registered for both groups using information from the municipality and by asking the families at baseline and at the 9- and 18-month follow-ups.

During the trial, all forms of intervention and concomitant care are allowed except for participation in any kind of ongoing and intensive family intervention program addressing parental functioning and child development involving all family members.

#### Outcomes

**Fig. 2:**
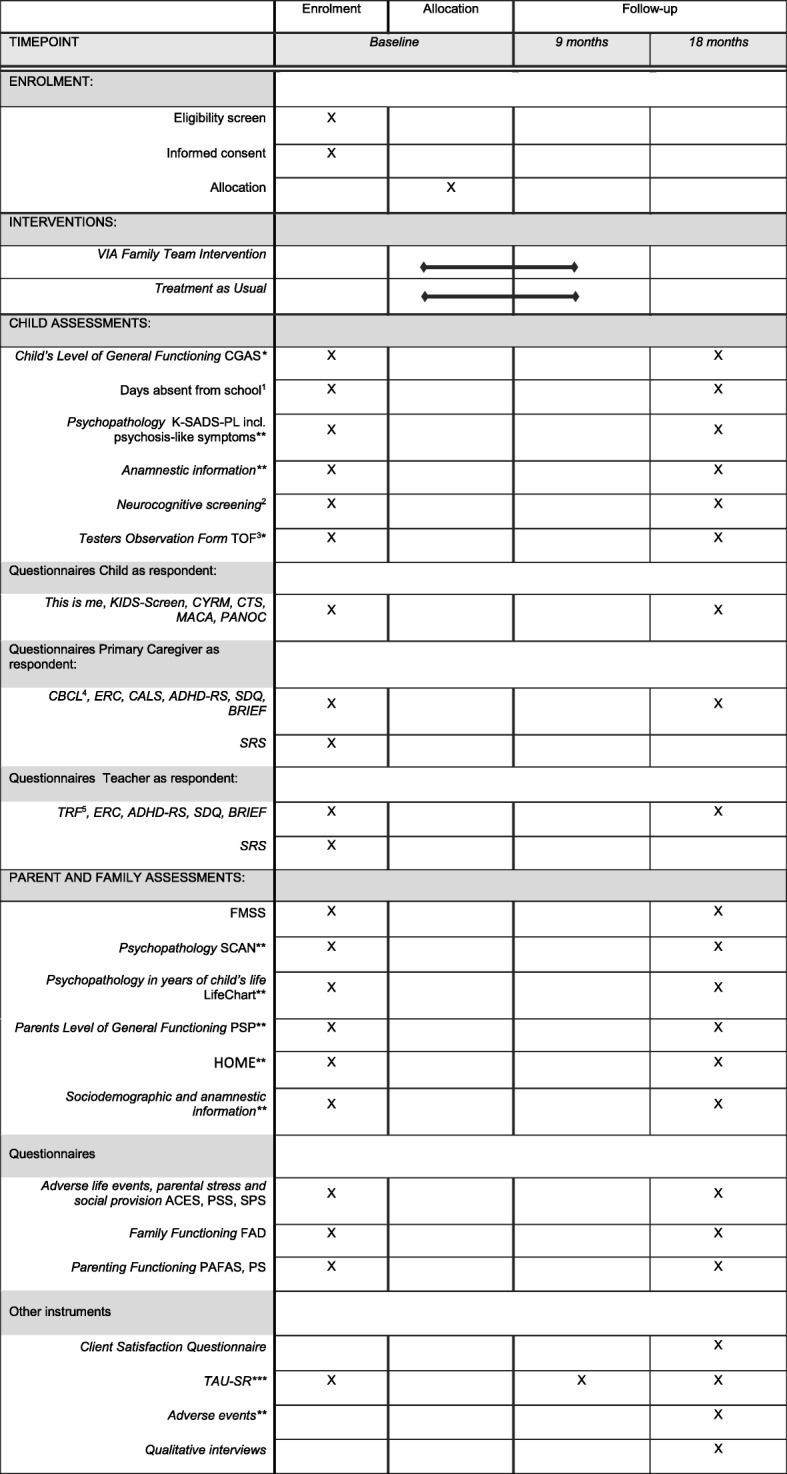
Schedule of enrolment, interventions, and assessments (SPIRIT Figure). *Clinical rating, **semi-structured interview, ***self-report on use of treatment and intervention facilities in private and public institutions (by any family member) ^1^ Days of absence from school (registry, parents report, and teachers report). ^2^ Neurocognitive tests: RIST; Wechsler Intelligence Scale for Children, 4th Edition (Coding); Rey Complex Figure Test and Recognition Trial; and Test of Memory and Learning, 2nd Edition (Memory of Stories). ^3^ Clinical Rating: TOF is in ASEBA. ^4^ CBCL is in ASEBA. ^5^ TRF is in ASEBA. ACES Adverse Childhood Experiences, ADHD-RS Attention Deficit Hyperactivity Disorder Rating Scale, ASEBA Achenbach System of Empirically Based Assessment, BRIEF Behavior Rating Inventory of Executive Function, CALS Children’s Affective Lability Scale, CBCL Child Behavior Checklist, CGAS Children’s Global Assessment Scale, CTS Childhood Trauma Screener, CYRM Child and Youth Resilience Measurement, ERC Emotion Regulation Checklist, FAD Family Assessment Device, FMSS Five-Minute Speech Sample, HOME Home Observation for Measurement of the Environment, K-SADS-PL Kiddie Schedule for Affective Disorders and Schizophrenia: Present and Lifetime, MACA Multidimensional Assessment of Caring Activities Checklist, PAFAS Parenting and Family Adjustment Scale, PANOC Positive and Negative Outcomes of Caring Questionnaire, PS Parenting Scale, PSP Personal and Social Performance Scale, PSS Parental Stress Scale, RIST Reynolds Intellectual Screening Test, SCAN Schedules for Clinical Assessment in Neuropsychiatry, SDQ Strengths and Difficulties Questionnaire, SPS Social Provision Scale, SRS Social Responsiveness Scale, TAU-SR Treatment as Usual Self Report, TOF Test Observation Forms

All outcome measures are listed in Fig. 2 for the 18-month follow up. The children can later be traced uniquely in the Danish registers, and information on e.g., the need for supportive efforts, placement out of home, and termination of elementary schooling can be investigated.

The selection of assessment instruments for the intervention outcomes is based upon results from the Danish High Risk and Resilience Study VIA 7 (conducted by the last author [49]), and focus group interviews conducted prior to the study with parents with schizophrenia and bipolar disorder who participated in the VIA 7 study. The same study confirmed that the instruments are relevant and acceptable for families to complete.

##### Primary outcome

The primary outcome is the change in the estimate of the child’s general functioning, as measured by the current score on the Children’s Global Assessment Scale (CGAS) [55]. It is an estimate of the child’s lowest level of functioning within the previous month. CGAS will be measured at baseline and after 18 months of the intervention. CGAS is a scale from 1 to 100 (the higher score, the better the function), which is included in the diagnostic interview Kiddie Schedule for Affective Disorders and Schizophrenia (Present and Lifetime; K-SADS-PL) [56]. The K-SADS-PL interview is done separately with the parent and the child, and covers all kinds of mental or psychiatric problems that a child can have. It also concerns the child’s daily level of functioning in the family, in school, and during leisure time, which is the basis of setting the CGAS score. CGAS is often used in research and considers all available information. It has been shown to have high validity and acceptable interrater reliability [57]. It is a dimensional and detailed measurement that accommodates that a given diagnosis (e.g., attention deficit hyperactivity disorder) may have a very different impact on the functioning of different children. The CGAS score will be rated by a group of trained and blinded clinicians with experience in child and adolescent mental health services and experience and training in the use of CGAS.

##### Secondary outcomes for the child

- *Change in extent of psychopathology.* Psychopathology is measured by the Child Behavior Checklist (CBCL) [58] after 18 months. The CBCL is a questionnaire with 113 items that is given to the parent. A score of 0 indicates that the child almost never displays the behavior (e.g., being impulsive), a 1 indicates that the behavior is sometimes present, and 2 that the behavior is observed often or always. A maximum (but not realistic score) is 226. Clinical cases, such as e.g., autism or attention deficit hyperactivity disorder, score approximately 60 on the CBCL scale. If the parents agree, the child's main teacher/pedagogue will also be asked to fill in the Teachers Report Form (same questions as the CBCL). The CBCL is very well validated and is widely used. It has the advantage of being dimensional and has been shown to have very good correlations with clinical diagnoses in numerous studies (http://www.aseba.org/ordering/reliabilityvalidity.html). Danish norms are available [59, 60].
- *Change in number of days absent from school within the last 6 months.* This is an objective proxy measure for the child’s well-being and family function. It assumes that children in families with poor functioning have a higher frequency of absence from school due to lack of support and structure in everyday routines, or due to more frequent somatic complaints resulting in the child staying home from school. It is mandatory for schools to report to the municipality days of absence for all pupils. Data will be collected for the 18 months of the intervention and changes will be analyzed.

##### Secondary outcomes for the family

- *Evaluation of family functioning.* This is assessed with the Family Assessment Device (FAD) [61]. FAD is a thorough questionnaire with 60 items based on a comprehensive sociological theory about the different functions of a family. It is completed by the parents or the actual caregivers in the family. It will be used at baseline and after 18 months.
- *Level of stimulation and support in the home.* This is evaluated by the Home Observation for Measurement of the Environment (HOME) [62], which is a semi-structured interview that can be done only in the home with both the child and the parent present. HOME is a continuous scale A score under the predefined cut-off indicates that there may be problems in the family.

#### Sample size

We used G*Power to calculate the sample size and power of the study [63, 64]. We assumed that the treatment and control groups were equal in size with the same standard deviation (SD). The SD for the sample size calculation was obtained from previous studies [30] and from a cross-national reliability study [65].

- *Primary outcome CGAS.* If the intervention results in an increase of the CGAS score of 10 points (e.g., from 55 to 65, SD = 13), which is a realistic estimate, we will have a high effect size. Power calculations show that by including 37 children in each group, we will be able to measure a difference of 10 points on the CGAS score between the two groups with a power of 0.90.
- *Secondary outcome CBCL.* If the intervention reduces the total problem score on CBCL by 10 points (from 28 to 18, SD =15), which will be clinically very relevant and realistic, we will have an effect size of 0.66. Power calculations show that with 37 in each group, we will be able to detect a difference between the two groups of 10 points in the CBCL with a power of 0.80.
- *Secondary outcome FAD*. In a previous study with a similar psychiatric population, the FAD general functioning score was normally distributed with SD = 0.51 (scale: 1–4, mean: 2.27) [66]. If the true difference between the mean of the VIA Family group and the mean in the TAU group at the 18-month follow-up is 0.4, we will need to study 35 families in each treatment group. Therefore, we need a total of 70 families to be able to reject the null hypothesis that the population means of the VIA Family and TAU groups are equal with probability (power) 0.90. The probability of a type I error associated with this test of this null hypothesis is 0.05.

#### Recruitment

##### Method for recruitment in the study

**Fig. 3:**
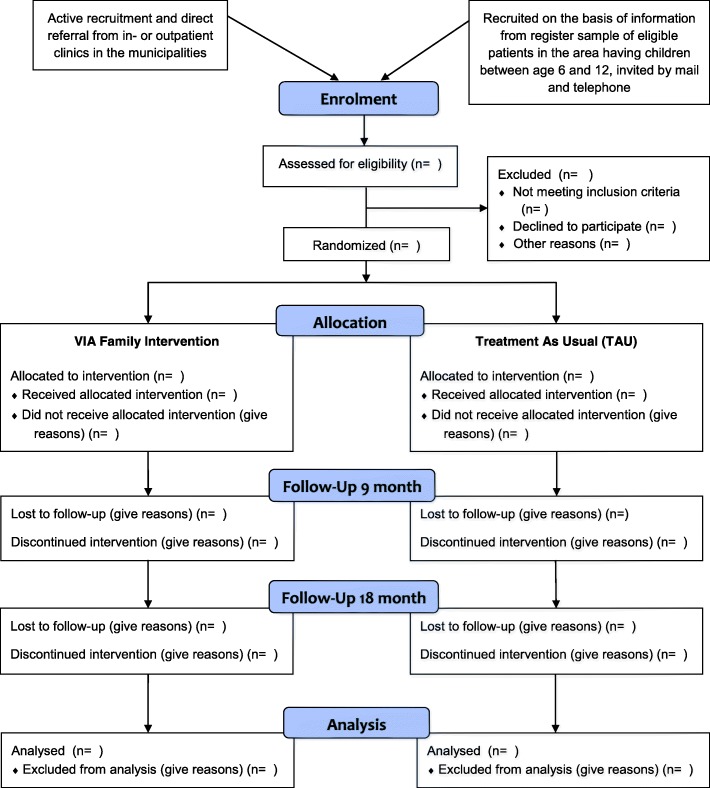
CONSORT flowchart for participants in the VIA Family study. TAU treatment as usual

A list of eligible participants will be drawn from the Danish Psychiatric Central Register and the Central Person Register and will, together with those referred directly from the in- or outpatient clinics in the Mental Health Center of Copenhagen, form the group of potential participants. At least 100 families will be invited to participate (Fig. 3). Data from the Danish registries show that a sufficient number of participants can be found within the catchment area of the trial, and that recruiting 100 families is realistic even if some potential participants decline.

Eligible parents and families will receive a letter and an e-mail in their personal inbox, which is used for public communications of all kinds in Denmark. They will receive a folder briefly describing the project and an invitation to participate in the study. After the initial mail, the research team will invite all families by phone to an information meeting about the study. Moreover, parents who fit the inclusion criteria can be directly referred to the study from in- or outpatient clinics in the municipalities.

If there are children in the family who are not aged between 6 and 12, they are included in the activities of the intervention, but the assessment prior to randomization and at follow-up will involve only children within the defined age group at baseline.

##### Procedure for recruitment and enrollment if parents do not live together

The ill parent does not need to live with the other parent or with the child if the child lives in the municipalities of Frederiksberg or Copenhagen. In these cases and when the parents share custody of the child, the first step is to ask the ill parent for permission to invite the child and the other parent. Both parents do not need to participate, but if there has been a divorce or legal separation with shared custody of the child, both parents need to give their informed consent for the child’s participation in the project.

### Allocation

Randomization is carried out by a member of the intervention team situated away from the research assessors. The team member assigns participants to interventions. The allocation concealment mechanism is performed by a computer algorithm saved in REDCap [54]. The randomization process cannot be influenced by the person who makes the randomization or any other person. The randomization sequence is set in a preset concealed block size order with an allocation ratio 1:1. The randomization is stratified solely by diagnostic group.

### Blinding

Outcome assessors, data analysts, and researchers will be blinded throughout the study, including during the statistical analysis. In addition, researchers are blinded to block size and frequencies. It is not possible to blind the participants nor those providing the intervention. The VIA Family intervention takes place at a site away from the assessors, data analysts, and researchers, which combined with the minimal interaction and communication between the intervention team and the assessors, aims to minimize the risk of unblinding. If unblinding occurs during the study, it will be registered and another assessor will perform the outcome assessment at the follow-up for the family whose treatment allocation has been revealed.

In theory, no circumstances should necessitate unblinding of the assessors or researchers, since all relevant information and clinical evaluations will be available to the research manager (AAET) and members of the intervention team, who can intervene in an emergency.

### Data collection, management, and analysis

#### Data collection methods

Families are assessed with a range of diagnostic interviews, neurocognitive tests, interviews, and questionnaires at baseline and at the 18-month follow-up (Fig. 2 and Table 1). The assessment at baseline takes approximately 8 h in total for a family of two adults and one child. The 9-month follow-up consists of a mid-phase telephone call by an independent research assistant. This contact seeks to decrease the risk of attrition from the study. Data will be collected from all participants independently of compliance to treatment.

**Table 1:** Test battery

Instrument Name	Domain	Child	Parent	Teacher
CGAS [55]	Daily functioning	x	x	
HOME [62]*	Home environment, level of stimulation, and support	x	x	
K-SADS-PL, including psychosis-like symptoms [56]	Diagnostic screening and psychopathology	x	x	
FMSS [72]	Parent–child relationship		x	
RIST [73]	General intelligence	x		
Coding from WISC-IV [74]	Processing speed	x		
RCFT [75]	Visual memory	x		
TOMAL-II: Memory for Stories [76]	Verbal memory	x		
This is me [77]	Self esteem	x		
Kids-Screen [78]	Quality of life	x		
CTS [79]	Childhood trauma	x		
CYRM [80]	Resilience	x		
MACA-YC18 + PANOC-YC20 [81]	Over-responsibility and caretaking	x		
CALS [82]	Affective lability		x	
CBCL [58]	Psychopathology		x	
TRF [83]	Psychopathology			x
BRIEF [84]	Executive functioning		x	x
ADHD-RS [85]	Attention		x	x
SDQ [86]	Daily functioning and psychopathology		x	x
SRS-2 [87]	Social cognition		x	x
ERC [88]	Emotion regulation		x	x
FAD [61]	Family functioning		x	
PSS [89]	Parental level of stress		x	
PAFAS [90]	Parental and family functioning		x	
Parenting Scale [91]	Parenting style		x	
SPS [92]	Social network, adult		x	
ACES [93]	Adverse life events, adult		x	
PSP [94]	Daily functioning, adult		x	
Anamnesis	Socioeconomic status, developmental milestones, adverse life events, school, and family relations		x	
SCAN interview, including LifeChart [50]	Psychopathology in adults and course of illness in relation to child’s age		x	
Absence from school, child			x	x
CSQ [95]	Client satisfaction	x	x	
TOF [96]	Psychopathology: clinical rating after 60 min. of neuropsychological testing	x		

ACES Adverse Childhood Experiences, ADHD-RS Attention Deficit Hyperactivity Disorder Rating Scale, BRIEF Behavior Rating Inventory of Executive Function, CALS Children’s Affective Lability Scale, CBCL Child Behavior Checklist, CGAS Children’s Global Assessment Scale, CSQ Client Satisfaction Questionnaire, CTS Childhood Trauma Screener, CYRM Child and Youth Resilience Measurement, ERC Emotion Regulation Checklist, FAD Family Assessment Device, FMSS Five-Minute Speech Sample, HOME Home Observation for Measurement of the Environment, K-SADS-PL Kiddie Schedule for Affective Disorders and Schizophrenia (Present and Lifetime), MACA-YC18 Multidimensional Assessment of Caring Activities Checklist for Young Carers 18 items, PAFAS Parenting and Family Adjustment Scale, PANOC-YC20 Positive and Negative Outcomes of Caring Questionnaire for Young Carers 20 items, PSP Personal and Social performance Scale, PSS Parental Stress Scale, RCFT Rey Complex Figure Test, RIST Reynolds Intellectual Screening Test, SCAN Schedules for Clinical Assessment in Neuropsychiatry, SDQ Strengths and Difficulties Questionnaire, SPS Social Provision Scale, SRS Social Responsiveness Scale, TOF Test Observation Form, TOMAL-II Test of Memory and Learning, 2nd Edition, TRF Teachers Report Form, WISC-IV Wechsler Intelligence Scale for Children, 4th Edition

*) For children living 50/50 with mother and father, the HOME interview will be made in both homes

To ensure the high quality of data, all assessors are certified in administering K-SADS-PL, SCAN, and HOME and perform regular co-ratings of videotaped interviews. Furthermore, assessors are trained in administering the Personal and Social Performance Scale (PSP) [67], and the CGAS and ratings are done in an expert group setting. Only trained clinical psychologists administer the neurocognitive tests. All diagnoses are confirmed at clinical conferences with a child and adolescent psychiatrist being present.

#### Data management

All data, including all personal information about potential and enrolled participants, are entered directly into the data entry system REDCap [54] by the assessors on site, except for a few neurocognitive tests that can only be made on paper. Self-report surveys for adults are either answered electronically on site or at home and in rare cases on paper if need be (e.g., by families who do not have access to the internet at home and wish to fill out the surveys at home). The surveys answered on paper are entered into the data entry system REDCap using double data entry.

All surveys for children are read out to the child. The reasons are (1) to ensure standard procedures are used, (2) to minimize the risk of variance between assessors’ judgement of children’s ability to read for themselves, and (3) to avoid having surveys read to younger children and not to older ones.

Range checks for data values have been implemented in the data entry systems in REDCap.

#### Statistical methods

All tests will be two-tailed. The primary outcome analysis will be by intention to treat. Multiple imputations will be used to handle missing data (if any). The imputations will be based on a linear regression model with 100 imputations and 20 iterations. Pooled analyses will subsequently be used for our analysis. In the imputation model, we will select possible variables if they are independent predictors of the outcome or predictors of missing values (*P* < 0.05 in a univariable model). Analysis of covariance (ANCOVA or MANOVA as appropriate) will be used to calculate any significant results between the two groups, using the baseline value and the sex of the child as stratification variables.

All distributions of continuous outcome variables will be assessed for normality using a visual inspection of histograms with a normal probability curve and Q–Q plots. If a variable is not normally distributed, the variable will be transformed (e.g., log or square root), and if unsuccessful, a non-parametric test (e.g., the Wilcoxon–Mann–Whitney test) will be used.

For dichotomous outcomes, we will perform multiple logistic regressions with TAU as the reference and stratification variables as covariates after having imputed missing values (if any) using a logistic regression model. If the experimental groups are not significantly correlated to the outcome (*P* > 0.05), no further analyses will be performed. If we find a significant correlation, a model that is equivalent to the approach for continuous variables will be used.

Sensitivity analyses will include an analysis of complete cases, removal of outliers (defined as standardized residuals greater than 3 standard deviations), a per protocol analysis defining participants not having a single contact as violating the protocol, and a second per protocol analysis including only participants attending at least 50% of intended personal meetings in the VIA Family group. The second per protocol analysis is likely to cause severe selection bias, as the VIA Family group would include the participants with the highest level of motivation. Therefore, it is considered meaningful to report only negative results from this analysis.

### Monitoring

The study does not have a data monitoring committee. All study participants randomized to VIA Family are monitored for harmful events by the intervention team or, in cases of hospitalization, by their general practitioner or a doctor from the Mental Health Center. If necessary, the principal investigator will make the final decision to terminate the trial. All study participants will be asked to report voluntarily all adverse events or other unintended effects of the trial at the 9-month and 18-month follow-ups. The study will be audited weekly by the principal investigator group, who are not involved in the day-to-day work of the study and are independent of the sponsors.

## Discussion

There is an urgent need for visionary strategies and preventive interventions in mental health services. Currently, we do not have a specific approach for preventing mental illness in children who are born with a well-documented familial-based increased risk. The VIA Family study is an innovative study attempting to investigate the effects of an early, family-based, flexible, and multidisciplinary intervention for families with children who are known to have a significantly increased risk for developing a mental illness later in life. Further, evidence from qualitative studies and experience from large clinical studies show that these families often struggle with everyday problems to a much greater extent than other families [5].

The intervention to be tested, VIA Family, is based on both evidence from the existing literature and on the very first results from another Danish study, the Danish High-risk and Resilience Study VIA 7 [49], as well as on clinical experience and information from focus group interviews with mentally ill parents and their co-parents. Evidence from the literature has demonstrated that the vulnerability of these children is detectable at a very early age [10, 19]. Research and clinical experience have documented the increased risk of trauma, adverse life experiences, and sub-optimal living conditions, including problems with parenting for the children, all factors that we know increase the risk of later mental illness [22]. Qualitative analyses confirm that a multidisciplinary approach and close collaboration between different units and sectors are essential and needed to meet these families’ unmet needs [68]. In this way, the prevention will help to solve problems that exist here and now.

One of the theories behind the project is developmental traumatology, which focuses on the severe impact that any childhood trauma and adverse life events may have on a child’s brain and mental development, their cognitive and social functioning, their stress regulation and reward mechanism etc. [69]. If we can reduce the total load of these environmental risk factors and stimulate potential resilience mechanisms, then hopefully we can reduce each child’s risk of falling ill or experiencing other negative life events.

We have also investigated the large body of literature on resilience and how this can be strengthened in children. Factors like secure attachments, a social network, being part of a community, having positive experiences, and finding meaning in life are mentioned as crucial factors for individuals with a high degree of resilience [70, 71] .

### Strengths

The intervention being offered to the families is very intensive and flexible, and this should increase our chances of getting conclusive results. It lasts for 18 months and aims to meet almost any kind of problem that the families may struggle with. It is more intensive than those in most other studies [36]. Moreover, 18 months is quite a long intervention period. The intervention does not focus on illness and disabilities but more on everyday life in a non-stigmatizing way and is, thus, expected to be acceptable for most families. That the intervention is more intensive and comprehensive than those in other studies may be experienced in a negative way by some. However, each element has been chosen based on qualitative interviews and very recent results from a Danish cohort study [30, 31]. No treatment elements will be introduced before the family approve them and find them relevant and motivating.

By using comprehensive Danish registries, we will be able to follow these children in the future and have long term follow-up results, since some outcomes may be in the distant future. The assessment battery is comprehensive and covers several areas of interest with validated tests. For many of the tests we have Danish norms.

The target groups of people with schizophrenia, bipolar disorder, or major depression were chosen because evidence of the genetic impact in these disorders is well documented. However, from the child’s perspective, the diagnoses may not matter very much. Instead, it is the impact on the parent that is relevant. Parents with diagnoses other than those included in our study may have similar problems or behavior, e.g., negative symptoms, like anhedonia, lack of energy, restlessness, and anxiety. If the results of our study are positive, the next step will be to include families where one of the parents has other serious mental problems, e.g., parents with personality disorders or with multiple diagnoses or complex co-morbidities including substance abuse, to test the generalizability.

### Limitations

We intend to include 100 families in the study, which, based on the power calculation of our primary outcomes, is a sufficient number of participants for the study to show an effect of the intervention. However, it may be that the intervention is effective at a lower although still clinically relevant level, which would need a larger sample size to be detected.

Moreover, the study design is limited by the absence of data from the pilot phase of the study, which could have contributed to the design and feasibility assessment of the intervention. Although experience from the pilot testing of the intervention and the instruments for assessment, as well as the feedback from the pilot families, were included in the intervention and overall study design, this knowledge cannot contribute to the study of its feasibility. Hence, this trial will provide data that can be used as pilot data for the development of a revised version of the intervention manual and for future studies in this field.

Also, we include children from three different diagnostic categories, although we believe from research evidence that the children born to parents with schizophrenia are the most vulnerable of the three. From the child’s point of view, the diagnosis of the parent may not mean much in terms of how the family is functioning on a daily level, although the genetic impact may be milder for those born to parents with affective disorders. In particular, recruitment of families where one of the parents is suffering from schizophrenia may be the most troublesome.

Another challenge in all intervention studies is how to avoid attrition, especially from the control group. We hope that the initial feedback from the researchers, the 9-month status contact, and the emphasis on the importance of participating in research will keep the families in the study.

The intensive intervention may sound positive, but we should be aware that this could also be experienced as overwhelming or over-involving for some participants. Due to this study’s pragmatic and flexible approach, we will not be able to identify which of the treatment elements was the most effectual. Further, it may be a problem that 18 months of intervention is too short for the relevant differences to be recognizable.

### Trial status

The current protocol is Version 4, December 2018. Recruitment will take place from September 2017 to March 2019.

## Additional files

Additional file 1: Completed SPIRIT checklist. (DOC 127 kb)

Additional file 3: World Health Organization Trial Registration Data Set. (DOCX 21 kb)

Additional file 2: Guide for all study participants on psychosocial help and support. (DOCX 24 kb)

## Abbreviations

ACES: Adverse Childhood Experiences
ADHD-RS: Attention Deficit Hyperactivity Disorder Rating Scale
ASEBA: Achenbach System of Empirically Based Assessment
BRIEF: Behavior Rating Inventory of Executive Function
CALS: Children’s Affective Lability Scale
CBCL: Child Behavior Checklist
CGAS: Children’s Global Assessment Scale
CSQ: Client Satisfaction Questionnaire
CTS: Childhood Trauma Screener
CYRM: Child and Youth Resilience Measurement
ERC: Emotion Regulation Checklist
FAD: Family Assessment Device
FMSS: Five-Minute Speech Sample
FORBOW: Families Overcoming Risks and Building Opportunities for Well-Being
HOME: Home Observation for Measurement of the Environment
K-SADS-PL: Kiddie Schedule for Affective Disorders and Schizophrenia: Present and Lifetime
MACA: Multidimensional Assessment of Caring Activities Checklist
PAFAS: Parenting and Family Adjustment Scale
PANOC: Positive and Negative Outcomes of Caring Questionnaire
PS: Parenting Scale
PSP: Personal and Social Performance Scale
PSS: Parental Stress Scale
RCFT: Rey Complex Figure Test
REDCap: Research Electronic Data Capture
RIST: Reynolds Intellectual Screening Test
SCAN: Schedules for Clinical Assessment in Neuropsychiatry
SD: Standard deviation
SDQ: Strengths and Difficulties Questionnaire
SMI: Severe mental illness
SPIRIT: Standard Protocol Items: Recommendations for Interventional Trials
SPS: Social Provision Scale
SRS: Social Responsiveness Scale
TAU: Treatment as usual
TAU-SR: Treatment as Usual Self Report
TOF: Test Observation Form
TOMAL-II: Test of Memory and Learning, 2nd Edition
TRF: Teachers Report Form
Triple P: Positive Parenting Program
WISC-IV: Wechsler Intelligence Scale for Children, 4th Edition
