# Supplementary material for: VIA Family—a family-based early intervention versus treatment as usual for familial high-risk children: a study protocol for a randomized clinical trial
Source: Trials. 2019 Feb 8;20:112. doi: 10.1186/s13063-019-3191-0 (PMC6368720; doi:10.1186/s13063-019-3191-0)
Supplement: Supplementary file 2 — World Health Organization Trial Registration Data Set. (DOCX 21 kb) [file 13063_2019_3191_MOESM2_ESM.docx]

| **Data category** | **Information**[**^32^**](http://www.spirit-statement.org/spirit-statement/references#32) |
| --- | --- |
| **Primary registry and trial identifying number** | ClinicalTrials.gov NCT03497663 |
| **Date of registration in primary registry** | 13^th^ of April 2018 |
| **Secondary identifying numbers** | H-17000450 (Regional Committee on Health Research Ethics (DNVK) for the Capital Region of Denmark) |
| **Source(s) of monetary or material support** | Research Unit at Child and Adolescent Mental Health Center, Capital Region, Denmark. |
| **Primary sponsor** | Danish Ministry of Health (Satspuljen) |
| **Secondary sponsor(s)** | TrygFonden, Municipality of Frederiksberg, Denmark |
| **Contact for public queries** | ADM [Anne.Dorothee.Mueller@regionh.dk](mailto:Anne.Dorothee.Mueller@regionh.dk), ICTG [Ida.Christine.Tholstrup.Gjoede@regionh.dk](mailto:Ida.Christine.Tholstrup.Gjoede@regionh.dk), AAET [Anne.Amalie.Elgaard.Thorup@regionh.dk](mailto:Anne.Amalie.Elgaard.Thorup@regionh.dk) |
| **Contact for scientific queries** | *ADM, ICTG, AAET*  Research Unit at Child and Adolescent Mental Health Center, Capital Region, Denmark. |
| **Public title** | VIA Family – family based early intervention versus treatment as usual for familial high-risk children |
| **Scientific title** | VIA Family – family based early intervention versus treatment as usual for familial high-risk children. *Can a multidisciplinary specialized intervention improve general functioning and decrease symptoms of psychopathology in familial high-risk children born to parents with severe mental illness?* |
| **Countries of recruitment** | Denmark |
| **Health condition(s) or problem(s) studied** | Familial high-risk children: children born to parents with schizophrenia spectrum disorders, bipolar affective disorder or major recurrent depression. |
| **Intervention(s)** | Active comparator: A specialized multidisciplinary family-based intervention: VIA Family intervention Comparator: Treatment As Usual that is available for the targeted participants in this study. |
| **Key inclusion and exclusion criteria** | Ages eligible for study: Families with children age 6-12 Sexes eligible for study: both Accepts healthy volunteers: no  Inclusion criteria: Families who have at least one child aged 6-12 with an address registered in the municipality of Frederiksberg or Copenhagen. Families living within the municipality of Frederiksberg are prioritized. Further, at least one of the parents must have a diagnosis of schizophrenia spectrum disorder, bipolar affective disorder or recurrent moderate or severe depression. The parent with a diagnosis must have had at least one in- or outpatient contact with the mental health system within the lifetime of the child.  Exclusion criteria: 1.Parents who do not speak and understand enough Danish to be able to give informed consent for their own participation and for the child’s participation.  2.If all family members are currently engaged in an intensive family intervention program addressing parental functioning and child development, they are excluded from the study. |
| **Study type** | Interventional Allocation: randomized Intervention model: parallel assignment Masking: single blind (data analysts, researchers, outcomes assessors) Primary purpose: prevention |
| **Date of first enrolment** | September 2017 |
| **Target sample size** | 100 families |
| **Recruitment status** | Recruiting |
| **Primary outcome(s)** | Change in daily functioning measured by the Children’s Global Assessment Scale (CGAS) (time frame 18 month) |
| **Key secondary outcomes** | *Change in extent of psychopathology. M*easured by the Child Behavior Checklist (CBCL) (Time frame: 18 month)  *Change in number of days absent from school within the last 6 months* (Time frame: 18 month)  *Evaluation of family functioning* by Family Assessment Device(FAD) (Time frame: 18 month)  *Change in level of stimulation and support in the home* is evaluated by the Home Observation for Measurement of the Environment (HOME) (Time frame: 18 month) |
