# Supplementary material for: VIA Family—a family-based early intervention versus treatment as usual for familial high-risk children: a study protocol for a randomized clinical trial
Source: Trials. 2019 Feb 8;20:112. doi: 10.1186/s13063-019-3191-0 (PMC6368720; doi:10.1186/s13063-019-3191-0)
Supplement: Supplementary file 3 — Guide for all study participants on psychosocial help and support. (DOCX 24 kb) [file 13063_2019_3191_MOESM3_ESM.docx]

**Additional file 3**

**Guide on psychosocial help and support**

Before randomization all families will receive a brief telephone call which includes general information about the existing available services for help and support for familial high risk children in Frederiksberg and Copenhagen. The procedure where this information is passed on to the parents will be standardized and the services mentioned to the families will therefore not vary according to their specific needs.

1: Telephone advice/support services.

Hotlines focusing on children and parents are offered by “Børns Vilkår” and “Psykiatrifonden”. Other hotlines are offered by “Depressionslinjen” and “Livslinjen”.

2: Support groups for children born to parents with mental illness.

Currently “Psykiatrifonden” and “Psych-Info” offer those services.

3. General services within the municipalities:

To get support and help concerning psychiatric and/or psychological problems for the adult or the child, the general practitioners are to be contacted. In case of school related problems the parent can get support from with the school psychologist (PPR) or social worker at the school.
